# Association of Urinary Biomarkers of Renal Tubular Injury with Cognitive Dysfunction in Older Patients with Chronic Kidney Disease: A Cross-Sectional Observational Study

**DOI:** 10.3390/brainsci13040551

**Published:** 2023-03-25

**Authors:** Yiwei Wang, Ling Hu, Difei Zhou, Xiaolei Chen, Leting Zhou

**Affiliations:** 1 Department of Anesthesiology, The Affiliated Wuxi People’s Hospital of Nanjing Medical University, Wuxi People’s Hospital, Wuxi Medical Center, Nanjing Medical University, Wuxi 214023, China; wywtian@njmu.edu.cn (Y.W.);; 2 Department of Internal Medicine, The Affiliated Wuxi People’s Hospital of Nanjing Medical University, Wuxi People’s Hospital, Wuxi Medical Center, Nanjing Medical University, Wuxi 214023, China; hll1165298726@163.com; 3 Department of Nursing, The Affiliated Wuxi People’s Hospital of Nanjing Medical University, Wuxi People’s Hospital, Wuxi Medical Center, Nanjing Medical University, Wuxi 214023, China

**Keywords:** β2-microglobulin, cognitive dysfunction, chronic kidney disease, urinary biomarkers of renal tubular injury, older patients, mitochondrial DNA

## Abstract

Epidemiological data suggest that individuals in all stages of chronic kidney disease (CKD) have higher risks of developing cognitive impairment. The relationship between CKD and cognition has been assessed exclusively using glomerular function markers; however, kidney tubule injury has not been assessed. We assessed the association between urinary biomarkers of renal tubular injury and cognitive dysfunction in older patients with CKD Stages 3–4. According to the Montreal Cognitive Assessment, participants were divided into cognitive dysfunction and control groups. Compared with the control group, the cognitive dysfunction group had significantly higher percentages of smokers, noticeably lower average education, and higher mitochondrial DNA (mtDNA) levels in the peripheral blood. Spearman correlation analysis showed that higher urine neutrophil gelatinase-associated lipocalin, kidney injury molecule-1, and beta-2 microglobulin (β2M) levels were significantly associated with lower cognitive scores. Multivariate logistic regression analysis showed that only increased urinary β2M levels were independently associated with cognitive worsening in CKD after adjusting for confounders. Logistic regression identified a promising role of urinary β2M combined with smoking and education for predicting cognitive impairment in CKD. Urinary β2M and cognitive function negatively correlated with mtDNA content, suggesting that mitochondrial dysfunction is a common pathophysiological mechanism linking CKD and cognitive dysfunction.

## 1. Introduction

Chronic kidney disease (CKD) is a complex and heterogeneous disease that continues to outpace clinical management and has been increasingly recognized as a significant health problem worldwide [1]. The global burden of CKD is rising, with estimated prevalence rates of 11–13% in the general population, gradually increasing to more than one-third of adults over the age of 65 years. The United Nations projects that the global population aged 65 years and older will triple from 0.5 billion in 2010 to 1.5 billion by 2050 [2]. In China, the proportion of older individuals (aged 65 years and older) was 17.9% in 2019 [3]. Global aging has far-reaching implications for the health care of older patients with CKD due to the rapidly increasing demand for medical services. Although studies support the notion that cognitive dysfunction begins in early kidney failure [4,5], cognitive impairment is still underdiagnosed, likely due to its subtle presentation and the lack of routine screening in older patients with CKD.

There is growing evidence that cognitive disorders can result in decreased adherence to medications and treatment, poor nutrition, reduced quality of life, a loss of independence, heavier caregiver burden, and premature mortality [6]. Therefore, the early identification of cognitive impairment is essential to achieve better surveillance and diagnosis and to develop effective prevention and treatment strategies for patients with CKD.

Prior studies evaluating the relationship between CKD and cognitive impairment have evaluated glomerular function and injury exclusively, largely utilizing the estimated glomerular filtration rate (eGFR) or albuminuria, markers of glomerular filtration, to assess kidney health [7]. However, the assessment of renal tubule injury is notably absent. Many urinary biomarkers of renal tubular injury have emerged, such as kidney injury molecule-1 (KIM-1), neutrophil gelatinase-associated lipocalin (NGAL), monocyte chemoattractant protein-1 (MCP-1), and beta-2 microglobulin (β2M), allowing for the clinical assessment of renal tubular health [8]. Compared to glomerular biomarkers, urinary biomarkers of renal tubular injury may enable early detection, the identification of the location of the injury, etiologic discernment, and prognostic prediction of CKD [9].

Our cross-sectional, observational study evaluated the relationship between common urine markers of renal tubular injury and cognitive impairment among older participants with CKD. These findings may provide some clues for improving disease detection and the identification of risk factors for cognitive impairment, early diagnosis of subclinical cognitive impairment, and prediction of adverse events in various clinical settings.

## 2. Materials and Methods

### 2.1. Study Population

This study was approved by the Chinese Clinical Trial Registry (ChiCTR) with a registration number of ChiCTR2200059887. For this observational study, we enrolled participants between April and September 2022 at the Wuxi People’s Hospital, affiliated with Nanjing Medical University. CKD was defined according to the criteria of the United States National Kidney Foundation Kidney Disease Outcomes Quality Initiative. The staging was defined by the eGFR based on the presence of kidney damage and the level of kidney function. The eGFR was calculated using creatinine or cystatin C and the CKD-Epidemiology Collaboration equation, as described previously [10]. Stages 3–4 were categorized as an eGFR of 15–59 mL/min/1.73 m^2^. The inclusion criteria were (1) age ≥ 60 years; (2) Stage 3–4 CKD; (3) proteinuria < 1 g/day; and (4) no active infection or bleeding three months before enrollment. The exclusion criteria were (1) the inability to provide informed consent; (2) illiteracy; (3) hearing or visual disability, which could affect cognitive assessment; (4) a history of chemotherapy or radiation therapy for any cancer; and (5) a history of malignancy, stroke, dementia, or other psychiatric or neurological diseases. All participants provided informed consent for inclusion before participating in the study. The study was conducted in accordance with the Declaration of Helsinki, and the protocol was approved by the Ethics Committee of the Wuxi People’s Hospital, affiliated with Nanjing Medical University (KY22017).

### 2.2. Procedure

All participants completed self-administered questionnaires in the presence of trained interviewers to collect clinical information, including age, sex, years of education, medical history (hypertension, diabetes mellitus, and coronary heart disease history), lifestyle factors (smoking and alcohol use), and mental health conditions. Blood and urine samples were collected from participants between 6:00 a.m. and 8:00 a.m. following overnight fasting and analyzed at a centralized laboratory. Blood pressure, body weight, and height were also measured. Each subject’s body mass index (BMI) was calculated by dividing body weight by height squared (kg/m^2^). The participants completed a battery of cognitive function tests between 2:00 p.m. and 4:00 p.m. using the Montreal Cognitive Assessment (MoCA). According to the cognitive assessment results, the participants were divided into a normal cognitive group and a cognitive impairment group, with 50 patients in each group.

### 2.3. Blood Sample Collection

Blood was collected in serum tubes (BD Vacutainer, Franklin Lakes, NJ, USA) and ethylenediaminetetraacetic acid plasma tubes (BD Vacutainer, Franklin Lakes, NJ, USA). The samples were centrifuged for 10 min at 3000× *g*, and the supernatant was collected, aliquoted into sterile cryovials, and stored at −80 °C until assayed. Serum low-density lipoprotein (LDL) levels were measured in a single central laboratory using standard methods. 

### 2.4. Renal Tubular Injury Marker Measurements

All urine specimens were centrifuged for 5 min at 1500× *g*, and the supernatant was collected, aliquoted into sterile cryovials, and stored at −80 °C until assayed. Levels of urinary KIM-1, NGAL, MCP-1, and β2M were measured. Concentrations of KIM-1, NGAL, MCP-1, and β2M were measured using commercially available enzyme-linked immunosorbent assays using test kits for KIM-1 (ZK-1911; ZhenKe, Shanghai, China), NGAL (ZK-3747; ZhenKe, Shanghai, China), MCP-1 (ab179886; Abcam, Boston, MA, USA), and β2M (EHB2M; Invitrogen, Carlsbad, SD, USA), respectively, according to the manufacturer’s instructions. Commercial ELISA kits were used for the detection of urinary KIM-1, NGAL, MCP-1, and β2M, which used their recombinant proteins as reference material for positive and cut-off calibrator controls, respectively.

### 2.5. Assessment of Cognitive Function

The MoCA was used to evaluate cognitive function [11]. The MoCA is a valid and standardized screening tool that takes approximately 10 min to complete and is highly sensitive in detecting mild cognitive impairment. The MoCA grades multiple domains of cognition by measuring short-term memory, visuospatial abilities, executive function, language (expression and comprehension), attention, concentration, working memory, and orientation to time and place. The level of education was adjusted by adding one point to the total score for participants with ≤12 years of formal education. The maximum possible score on the MoCA is 30, with higher scores indicating better cognitive function. Cognitive function was dichotomized at 26 points on the MoCA. Participants with a score of <26 or those unable to complete the MoCA were classified as having cognitive impairment. Participants with a score of ≥26 were classified as not having cognitive impairment [12]. Then, the test score was used as a continuous variable in the analyses. The MoCA was administered and scored by a research coordinator following Mandarin version 7.1 instructions available at www.mocatest.org.

### 2.6. Mitochondrial Deoxyribonucleic Acid (mtDNA) Analysis

Total DNA was extracted from the serum using the TIANamp Genomic DNA Kit (TIANGEN, Beijing, China). Quantitative real-time polymerase chain reaction (PCR) was used to determine the mtDNA copy number according to the method developed by Wong et al. [13]. mtDNA was amplified using primers specific to the mitochondrial cytochrome B gene to generate a standard curve. The mtDNA copy number was normalized to the nuclear DNA copy number by amplifying the acidic ribosomal phosphoprotein P0 nuclear gene. Primer sequences were designed using Primer Premier 5.0 (PitchBook, San Francisco, CA, USA). The primers used were the following: forward 5′-GCCTGCCTGATCCTCCAAAT-3′ and reverse 5′-AAGGTAGCGGATGATTCAGCC-3′. The primers for 36B4 were forward 5′-AGGATATGGGATTCGGTCTCTTC-3′ and reverse 5′-TCATCCTGCTTAAGTGAACAAACT-3’. PCR was performed using a Rotor-Gene real-time centrifugal DNA amplification system (Corbett Research, Sydney, Australia) with the SYBR Green master mix (Applied Biosystems, Foster City, CA, USA). The thermal cycling conditions were the following: one cycle of 50 °C for 2 min and 95 °C for 15 min, followed by 35 cycles of 94 °C for 20 s, 56 °C for 30 s, and 72 °C for 30 s. The melting reaction was measured with a decrease of 1 °C per cycle between 72 and 92 °C. The mtDNA copy number was calculated using the formula described by Ryang et al. [14], and the cycle threshold values in each quantitative polymerase chain reaction were used to measure the copy number of mtDNA using standard regression analyses.

### 2.7. Statistical Analysis

The biomarkers were log_2_ transformed to minimize skew-biased data. Continuous data were represented as either mean ± standard deviation (SD) or median with interquartile range, depending on normality, which was assessed using the Shapiro–Wilk test. For two independent groups, the means of normally distributed data were compared using *t*-tests, and the medians of non-normally distributed data were compared using the Wilcoxon test. Categorical variables are expressed as numbers (percentages). The Chi-square test was used to test the relationship between two categorical variables. Correlation and multivariate logistic regression analyses adjusted for potential confounding variables were performed to assess the association between biomarkers of renal tubular injury and cognitive impairment. Receiver operating characteristic (ROC) curve analysis was performed to investigate the diagnostic power and optimal cut-off points for biomarkers of renal tubular injury using the area under the curve (AUC) and Youden index, respectively. Significance was defined as *p* < 0.05 for all analyses, and all reported *p*-values were two-sided. Statistical analyses were performed using R, version 3.6.2 (R Core Team, Vienna, Austria).

## 3. Results

### 3.1. Characteristics of the Study Population

In total, 50 patients (33 male and 17 female) with cognitive impairment (mean age 76.0 ± 7.2 years) and 50 individuals (38 male, 12 female) with normal cognition (mean age 74.0 ± 7.9 years) participated in this study. No differences in age, sex, BMI, drinking history, the number of hypertension medications, rates of diabetes, coronary heart disease, LDL values, or eGFR values were observed between the two groups. Table 1 shows the demographic and medical characteristics of the study participants. Years of education were lower among patients with cognitive impairment than among the control participants (*p* < 0.001, Table 1). There was a greater percentage of smokers among patients with cognitive impairment than among control participants (*p* < 0.001, Table 1). The data range of basic variables of study population was evaluated (Val min-Val maximum) in the Supplementary Material (Table S1).

### 3.2. Comparison of Urinary Biomarkers of Renal Tubular Injury in the Two Groups of Study Participants

**Figure 1:**
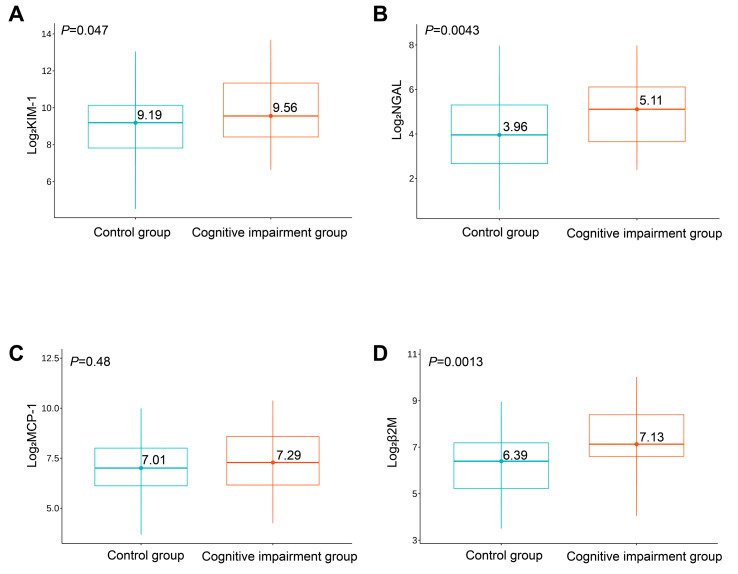
Expression levels of log2KIM-1 (**A**), log2NGAL (**B**), log2MCP-1 (**C**), and log2β2M (**D**) between the two groups of study participants.

Significantly increased urinary levels of log2KIM-1 (*p* = 0.047), log2NGAL (*p* = 0.004), and log2β2M (*p* = 0.001) were observed in the cognitive impairment group compared with the control group, while levels of log2MCP-1 (*p* = 0.48) did not significantly differ between the groups (Figure 1).

### 3.3. Association between Urinary Biomarkers of Renal Tubular Injury and Cognitive Impairment

**Figure 2:**
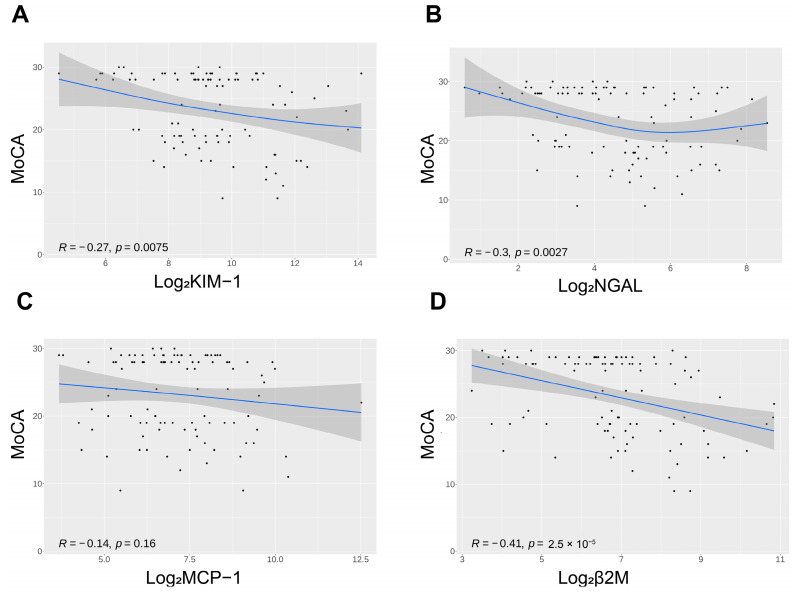
Spearman correlation analysis of KIM-1 (**A**), NGAL (**B**), MCP-1 (**C**), β2M (**D**), and MoCA scores.

When evaluating the unadjusted association between biomarkers of renal tubular health and cognitive function, measured using MoCA as a continuous variable using Spearman’s correlation, we found that higher urine KIM-1 (R = −0.27, *p* = 0.0075), NGAL (R = −0.30, *p* = 0.0027) and β2M (R = −0.41, *p* = 2.5 × 10^−5^) were significantly associated with lower cognitive scores but not with MCP-1 (R = −0.14, *p* = 0.16) (Figure 2). 

**Figure 3:**
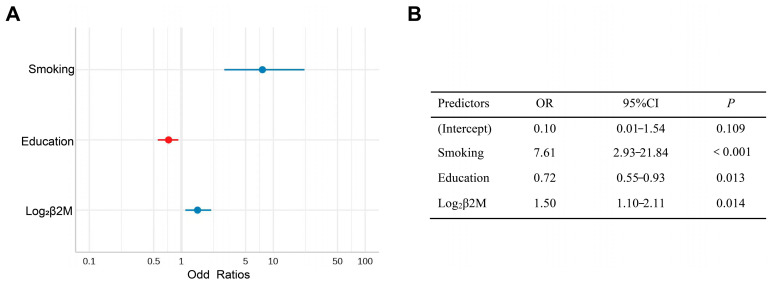
(**A**) Forest plot of the Multivariate logistics regression of risk factors for cognitive impairment. (**B**) Multivariate logistic regression analysis of risk factors for cognitive impairment.

In the univariate logistic analysis, education (odds ratio = 0.69, *p* = 0.001), smoking odds ratio = 6.05, *p* < 0.001), and three tubular injury markers (log_2_KIM-1: odds ratio = 1.31, *p* = 0.02; log_2_NGAL: odds ratio = 1.43, *p* = 0.005; log_2_β2M: odds ratio = 1.51, *p* = 0.002) were associated with the onset of cognitive dysfunction (Supplementary Material, Table S2). We further examined this association using multivariate logistic regression analysis. However, only higher β2M levels (odds ratio = 1.5, 95% confidence interval = 1.10–2.11, *p* = 0.014) remained significantly associated with a worse cognitive performance after adjusting for education and smoking, while NGAL and KIM-1 failed to show significance in the multivariable analysis (Figure 3).

### 3.4. ROC Curve Analysis of Single Urinary Biomarkers of Renal Tubular Injury and the Multivariable Model

**Figure 4:**
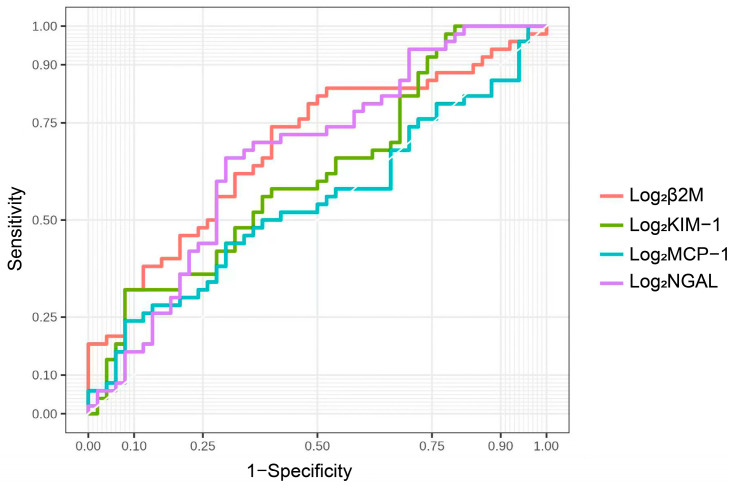
ROC curve analysis of single urinary biomarkers of renal tubular injury.

**Figure 5:**
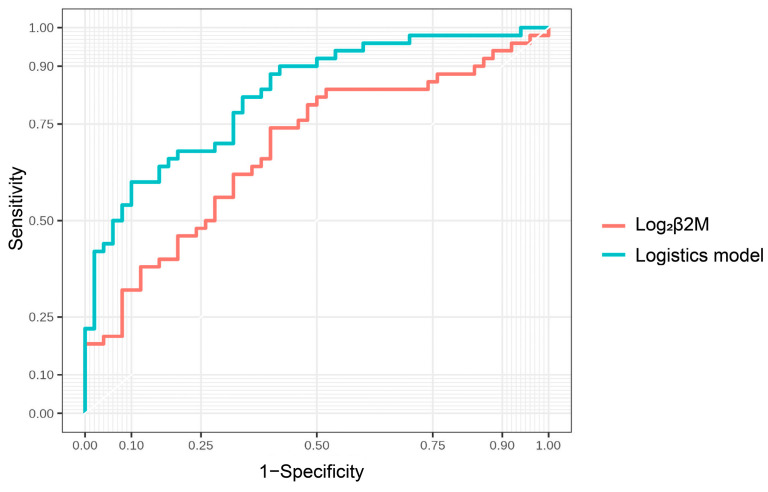
ROC curve analysis of the logistic regression model.

The ROC curve analysis revealed that urinary β2M levels were better predictors of cognitive impairment in patients with CKD than urine NGAL and KIM-1 levels (AUC = 0.69 for β2M, 0.67 for NGAL, 0.54 for NGAL, and 0.62 for KIM-1, respectively). Based on the ROC curve analysis, the best cut-off values for predicting cognitive impairment were 6.62 for log_2_ urinary β2M, yielding a sensitivity and specificity of 74% and 60%, respectively (Figure 4). A logistic regression model combining urinary β2M, education, and smoking was an optimal model for predicting cognitive impairment. An AUC of 0.83 was observed to predict cognitive impairment using this model, with a sensitivity and specificity of 60% and 90%, respectively (Figure 5). The AUCs of two ROC curves were compared using the DeLong test. The AUC of the logistic regression model was significantly increased compared with that of urinary β2-MG alone (*p* = 0.006), indicating that the diagnostic power can be improved by adding easy-to-obtain clinical characteristics (education and smoking).

### 3.5. Association of mtDNA Levels in Peripheral Blood with Tubular Injury Markers and Cognitive Impairment

**Figure 6:**
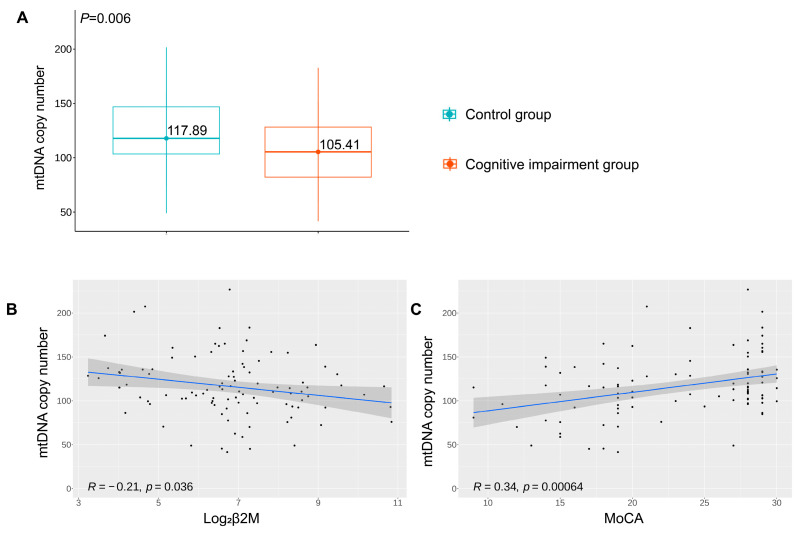
(**A**) mtDNA copy content in peripheral blood between the two groups of study participants. (**B**) Spearman correlation analysis of mtDNA content and log_2_β2M. (**C**) Spearman correlation analysis of mtDNA content and MoCA scores.

mtDNA content, measured as the amount of mitochondrial DNA copy number, was evaluated to assess mitochondria status. The amount of mtDNA was lower in the cognitive impairment group than in the control group (*p* = 0.006, Figure 6A).

To provide more insight into the relationship between renal tubule dysfunction and mitochondrial impairment, a correlation analysis between urinary β2M and mtDNA content was performed. It was suggested that urinary β2M levels negatively correlated with mtDNA content in a statistically significant manner (R = −0.21, *p* = 0.036, Figure 6B). We also attempted to examine the correlation between mtDNA content and cognitive impairment. The loss of mtDNA content was positively correlated with cognitive decline, measured as the MoCA score (R = 0.34, *p* < 0.001, Figure 6C).

## 4. Discussion

The prevalence of CKD has increased dramatically in older adults, from 11.13% in the general population to nearly 40% in individuals aged ≥ 60 years, and this may increase even further in the future [15]. Among older adults with CKD, approximately 17.5% have Stage 1–2 CKD, 75% have Stage 3 CKD, and 12.5% have Stage 4–5 CKD [16]. CKD is predicted to be the fifth-leading cause of death worldwide by 2040 [17]. Moreover, the prevalence and degree of cognitive decline increase with advanced CKD stages. When CKD progresses from Stage 3 to Stage 5, the prevalence of cognitive impairment among patients increases from 20–50% to 70% [18]. Studies examining the relationship between CKD and cognitive function have focused largely on hemodialysis patients, and little is known about cognitive dysfunction in pre-dialysis Stage 3–4 CKD. Because these conditions are often detected too late in the CKD course, no effective treatments have been developed to minimize cognitive impairment, alter the course of CKD, or limit the associated morbidity and mortality.

Epidemiological data suggest that CKD is strongly associated with cognitive impairment, and this association worsens with deteriorating renal function [19,20]. However, some studies have shown no association between CKD and cognitive function. These studies have mostly focused on glomerular function and injury, leading to mixed results. It is well known that CKD is not limited to the glomerulus. Instead, based on kidney biopsy, tubular atrophy and tubulointerstitial fibrosis are common findings in virtually all forms of CKD, and their severities have consistently proven to be reliable features for predicting the progression to end-stage kidney disease [21]. 

Tubular epithelial cells are the main constituent cells of the kidney and are highly sensitive to ischemia, hypoxia, poisoning, and other injury factors. Serum creatinine levels rise in the acute kidney injury (AKI) course after 24 h, limiting the ability for early detection and intervention in these cases. Biomarkers of renal tubular injury, including NGAL and KIM-1, measured within 4–6 h following AKI, have been demonstrated to predict the risk of AKI well before a rise in serum creatinine [8]. A study of over 1200 biopsies from donor candidates with a healthy kidney showed that tubulointerstitial fibrosis was present in 28% of patients, ranging from 3% in the 20–29 age group to 73% in those aged 70–79 years [22].

Previous studies might have underestimated the extent of the relationship between kidney disease and cognitive impairment. To address this evidence gap, we conducted a cross-sectional analysis of an observational study to determine the relationship between urinary biomarkers of renal tubular injury and cognitive function in patients with Stage 3–4 CKD at the Wuxi People’s Hospital, affiliated with Nanjing Medical University. Important exclusion criteria included proteinuria of >1 g/day to reduce the influence of damage to the glomerular filtration membrane. In the univariate analysis, NGAL, KIM-1, and β2M levels were negatively correlated with cognitive function. However, only higher β2M levels remained significantly associated with poorer cognitive function after adjustment for confounders in the multivariate logistic regression analysis.

β2M is an endogenous low-molecular-weight protein that easily passes through the glomerular filtration membrane and is almost entirely reabsorbed and degraded by proximal tubular cells, with less excretion in the urine [23]. Bianchi et al. reported that β2M is a better endogenous marker of GFR than serum creatinine [24]. β2M is a part of the histocompatibility leukocyte antigen complex on the cell membranes of all nucleated cells that synthesize it. It is released into circulation at a constant rate in normal subjects during normal cell turnover. β2M is freely filtered in the glomerulus before being readily reabsorbed in the proximal tubules, so urinary excretion is low in healthy individuals [23]. Thus, urinary β2M levels can be significantly elevated in cases of reabsorption dysfunction of the proximal renal tubules.

Our study demonstrated that urinary β2-MG levels in older patients with CKD and cognitive dysfunction were significantly increased and were negatively correlated with the MoCA score. The observed associations were independent of smoking and years of education. The AUC of the ROC curves was examined for each of the biomarkers of renal tubular injury for their ability to predict cognitive impairment in patients with CKD. The present study demonstrated that urinary β2-MG levels have a better diagnostic value than urine NGAL and KIM-1 levels. Logistic regression modeling identified the combination of urinary β2-MG, smoking, and education as optimal predictors for cognitive impairment. The addition of education and smoking to the model could be because of the observation that they were significantly different between the two groups with respect to the demographic characteristics of our study participants. Adding them to the model may correct this effect. The AUC of the logistic regression model was higher than that of urinary β2-MG only, indicating that the diagnostic power can be improved by adding easy-to-obtain clinical characteristics (education and smoking).

We believe that the early identification of cognitive impairment is important to optimize the compliance of patients with CKD, improve quality of life, and minimize premature death. This can be accomplished clinically by changing the care approach in practical ways to slow the progression of impaired cognition. Such strategies may include the optimal use of antiplatelet therapy and statins, meticulous blood pressure control, improved diet, exercise, cognitive stimulation, and retraining.

The mechanisms underlying the association between renal tubular health and cognitive impairment are uncertain and require further studies. The cognitive complications of CKD may be linked to an aberrant “kidney–brain axis”. Recent evidence suggests that there is a crosstalk between the kidney and brain and that this “kidney–brain axis” is sensitive to mitochondrial dysfunction, chronic inflammatory stress, and other mechanisms that promote vascular aging, which may lead to end-organ damage that is manifested clinically by the high prevalence of cognitive impairment observed during the progression of CKD [25]. This action of the proximal tubules is highly dependent on mitochondrial activity [26]. In the central nervous system, sufficient energy supply, which is required for neuronal survival and excitability, is mostly dependent on mitochondrial sources. Therefore, the brain is much more vulnerable to mitochondrial dysfunction [27]. Shlipak et al. proposed that mitochondrial dysfunction may be particularly important in defining the mechanisms linking kidney disease and cognitive dysfunction [28].

Mitochondrial respiratory chain dysfunction has been reported to be associated with mtDNA abnormalities. Malik and Czajka proposed that the mtDNA content can be a marker of mitochondrial dysfunction [29]. The premise of this theory is that the origins of mitochondrial impartment can be mutations in genes of nuclear DNA encoding mitochondrial proteins or in mtDNA. In contrast to nuclear DNA, mtDNA is more susceptible to damage, such as oxidative stress. In the initial presence of oxidative stress, reactive oxygen species contribute to mitochondrial biogenesis, resulting in an increased ratio between mtDNA and nuclear DNA. However, persistent oxidative stress may lead to the depletion of mtDNA, resulting from damaged mtDNA and proteins. The accumulation of damaged mtDNA may directly contribute to mitochondrial dysfunction playing a significant role in aging, increasing the cognitive decline that occurs with aging and aging-related neurodegeneration [30]. Therefore, according to this hypothesis, mtDNA content may precede mitochondrial dysfunction as an adaptive response and can therefore be a predictive marker.

In our study, we attempted a correlation between mtDNA content and cognitive decline, measured as the MoCA score. We found the loss of mtDNA content positively correlated with cognitive decline. Consistent evidence demonstrates that damaged mtDNA is linked to several mitochondrial disorders that have neurologic or cognitive sequelae. In a case-series report, Molnar et al. identified a general pattern of moderate-to-severe cognitive dysfunction across 19 patients with primary mtDNA mutations [31]. Lee et al. found that the mtDNA copy number in peripheral blood is associated with cognitive function in apparently healthy elderly women, which suggests that reduced mtDNA content may be a possible early marker of dementia [32]. It was observed that participants in the high mtDNA copy number group were more likely to have cognitive dysfunction than participants in the low mtDNA copy number group, which reinforced the results that the mtDNA copy number may be useful for monitoring the cognitive decline in older adults [33]. Prado found that circulating mtDNA levels may serve as a potential biomarker to determine the cognitive status of patients with schizophrenia. In this case, they assumed that the importance of mtDNA resides in its predisposition to the accumulation of mutations, capacity to trigger a pro-inflammatory state, and apoptosis, leading to cognitive impairment [34].

In addition, we found that urinary β2M levels negatively correlated with mtDNA content and that cognitive decline positively correlated with mtDNA content. These results suggest that reabsorption dysfunction in the proximal renal tubules and cognitive impairment may share the common pathology of mitochondrial dysfunction. However, the conclusions are hypothesis-generating, and further studies using a larger sample size are required to validate these results.

This study has a few limitations. First, this study only included older patients with Stage 3–4 CKD; therefore, the association between urinary β2M and cognitive impairment cannot be generalized to all patients with CKD, which requires a multi-center, large-sample, cohort study. Second, the present analysis reports a cross-sectional association between urinary β2M levels and cognitive impairment. As such, a causal association cannot be established, and prospective studies should further confirm the conclusions.

## 5. Conclusions

Urinary β2-MG, in combination with education and smoking, has the potential to identify cognitive dysfunction in older patients with CKD. Mitochondrial dysfunction may be a common pathophysiological mechanism that links CKD and cognitive dysfunction. Further studies are needed to elucidate this phenomenon and its underlying mechanisms in the context of its potential therapeutic value.

## Figures and Tables

**Table 1:** Characteristics of the study population.

Variables	Control Group	Cognitive Impairment Group	*p*
Sex (male) ^‡^	33 (66%)	38 (76%)	0.40
Age(years) ^§^	76 (7.2)	74 (7.9)	>0.90
BMI (kg/m^2^) ^†^	24.52 (23.22, 25.82)	24.52 (23.22, 25.82)	0.50
Education (years)^†^	6.00 (4.00, 7.00)	4.00 (3.00, 5.75)	<0.001
Smoking ^‡^	16 (32%)	37 (74%)	<0.001
Drinking ^‡^	14 (28%)	22 (44%)	0.14
Hypertension ^‡^	30 (60%)	28 (56%)	0.80
Diabetes ^‡^	19 (38%)	16 (32%)	0.70
Coronary Heart Disease ^‡^	16 (32%)	14 (28%)	0.80
LDL (mmol/L) ^†^	2.95 (2.77, 3.08)	2.84 (2.67, 3.06)	0.10
eGFR (mL/min/1.73 m^2^) ^†^	46 (42, 52)	44 (40, 49)	0.051
MoCA (points) ^†^	28.0 (28.0, 29.0)	19.0 (15.0, 20.0)	<0.001

Note: BMI, body mass index; LDL, low-density lipoprotein; eGFR, estimated glomerular filtration rate; MoCA, Montreal cognitive assessment; ^†^ Wilcoxon test; ^‡^ Chi-square test; ^§^ *t*-test; *p* < 0.05 for significance.

## Data Availability

The data presented in this study are available on reasonable request from the corresponding author. The data are not publicly available due to privacy.

## Supplementary Materials

The following supporting information can be downloaded at https://www.mdpi.com/article/10.3390/brainsci13040551/s1: Table S1: Data range of basic variables of study population; Table S2: Univariate logistic analysis of risk factors of cognitive impairment.
