# Supplementary material for: Association of Urinary Biomarkers of Renal Tubular Injury with Cognitive Dysfunction in Older Patients with Chronic Kidney Disease: A Cross-Sectional Observational Study
Source: Brain Sci. 2023 Mar 25;13(4):551. doi: 10.3390/brainsci13040551 (PMC10136492; doi:10.3390/brainsci13040551)
Supplement: Supplementary file 1 [file brainsci-13-00551-s001.zip › brainsci-2206906-supplementary.pdf]

Table S1. Univariate logistic analysis of risk factors of cognitive impairment.

	OR	95%CI	*P*
Education	0.69	0.55-0.86	0.001
Smoking	6.05	2.60-14.85	<0.001
Log_2KIM-1	1.31	1.05-1.68	0.020
Log_2NGAL	1.43	1.24-1.86	0.005
Log_2β2M	1.51	1.17-2.02	0.002

Table S2. Data range of basic variables of study population

Variables	Control group (Val min-Val maximum)	Cognitive impairment group (Val min-Val maximum)
Age(years)	60-94	62-90
BMI (kg/m^2)	21.58-29.44	19.79-29.92
Education (years)	2-15	1-10
LDL (mmol/L)	2.38-3.90	2.28-4.25
eGFR ($mL/min/1.73m^2$)	22.80-59.81	20.31-59.14
MoCA (points)	26-30	9-25
